# From P-Values to Objective Probabilities in Assessing Medical Treatments

**DOI:** 10.1371/journal.pone.0142132

**Published:** 2015-11-24

**Authors:** David Kault, Sam Kault

**Affiliations:** 1 Discipline of Mathematics, College of Science, Technology and Engineering, James Cook University, Townsville, Australia; 2 Centre for Applied Health Economics, Menzies Health Institute, Griffith University, Brisbane, Australia; Swiss Federal Institute of Technology (ETH Zurich), SWITZERLAND

## Abstract

The assessment of the effectiveness of a treatment in a clinical trial, depends on calculating p-values. However, p-values are only indirect and partial indicators of a genuine effect. Particularly in situations where publication bias is very likely, assessment using a p-value of 0.05 may not be sufficiently cautious. In other situations it seems reasonable to believe that assessment based on p-values may be unduly conservative. Assessments could be improved by using prior information. This implies using a Bayesian approach to take account of prior probability. However, the use of prior information in the form of expert opinion can allow bias. A method is given here that applies to assessments already included or likely to be included in the Cochrane Collaboration, excluding those reviews concerning new drugs. This method uses prior information and a Bayesian approach, but the prior information comes not from expert opinion but simply from the distribution of effectiveness apparent in a random sample of summary statistics in the Cochrane Collaboration. The method takes certain types of summary statistics and their confidence intervals and with the help of a graph, translates this into probabilities that the treatments being trialled are effective.

## Introduction

Evidence Based Medicine (EBM) is the dominant paradigm in assessing the effectiveness of clinical treatments. Conventionally much weight is given to analysis of clinical trials and in particular to whether p-values are more than or less than 0.05. Effectiveness then, is largely judged by whether a certain coincidence may or may not explain suggestive results. Researchers may not always be fully conscious of the tenuous basis for these decisions [1]. However this approach to assessing medical treatments has had a number of benefits:

- It has revealed a small number of treatments where mechanistic reasoning predicted effectiveness, but where clinical trials have subsequently shown surprisingly, that the treatment was in fact counterproductive. Indeed, an instance of this sort regarding the management of heart attack with lignocaine provided a major stimulus to the adoption of EBM. [2]
- The EBM position of accepting that there is appreciable evidence of effect only if p≤0.05 and at least provisionally ignoring mildly favourable results, is inherently a conservative position. This conservatism is a reasonable antidote to the presence of publication bias towards positive results, perhaps enhanced by commercial pressure [3, 4] and even fraud [5] in the case of new drugs, together with perhaps an undue enthusiasm for new treatments with poorly defined benefits and unknown dangers. A conservative approach will also be appropriate in situations where there are a large number of speculative associations with the number of false associations likely to greatly outweigh the number of true associations. [6]
- A philosophical position that discounts treatments which have not passed the p≤0.05 test, also permits an ethical position of equipoise thereby sanctioning the use of a placebo arm in clinical trials of promising but unproven treatments. [7]
- Finally, p-values as a criterion for the assessment of medical treatments, have the important benefit of being objective as they rely only on calculations from the data.

On the other hand there are important limitations to p-values based assessment of medical treatments:

- Though assessments of effectiveness based almost solely on p-values, continues to be the norm in practice [8], numerous statisticians and medical researchers have emphasised that a p-value should be only one of the ingredients in the assessment of medical treatments [9–12] and indeed, this position is now accepted to a degree within EBM [13].
- A p-value criterion of 0.05 seems too conservative when clinical trials are used to assess established treatments or treatments backed by mechanistic reasoning. Assume such trials specify a Type I error of 0.05 and a Type II error of 0.2 for detecting an appropriate level of effectiveness. Further assume that all treatments are either totally ineffective or are effective at the minimum levels for which the trials are designed. If in such situations, the proportion of effective treatments is 50%, then the false positives will constitute less than 6% of all positive results and Type II errors will occur four times more often than Type I errors. In such settings the p-value = 0.05 criterion seems too conservative. Interestingly, whilst the calculations in this paper generally agree, they also show unexpected situations where a p-value of 0.05 may not be conservative enough.
- A decision theory analysis of a treatment taking into account costs of errors, will require a probability of effectiveness, not a p-value.
- The p-value as a criterion is also unsatisfactory as it is easily misinterpreted by a naive reader, unlike a statement such as “researchers are *x*% sure the new treatment is effective”. Indeed confusion between p-values and “the chance that it is chance” seems widespread [1].

Bayesian statistics addresses some of the limitations of basing assessment of treatments largely on p-values, by directly calculating a probability that a treatment is effective. However, calculation of the probability that a treatment is effective in the light of the data, the posterior probability, requires as a starting point, the probability that the treatment will be effective prior to seeing the data. This reliance on a prior probability often involves using expert opinion, but this may be quite subjective and indeed sometimes unduly influenced by commercial pressures [14]. With objectivity almost inherent in the term “EBM”, this may be the deciding reason for EBM not embracing the Bayesian approach [1].

This paper gives a method of obtaining prior probabilities for a Bayesian approach in a way which is objective, but which also adds useful prior information to the assessment of medical treatments. No subjective assessment about the treatment being trialled is required. Instead calculation of the prior probability relies only on an estimate of the distribution of effectiveness apparent in summary statistics published in the Cochrane Collaboration, a compendium of EBM [15]. The distribution of summary statistics in the Cochrane Collaboration is assessed using a random sample from this compendium, with the proviso that studies concerning drugs under patent are excluded.

The distribution of effectiveness is assumed to fit a model. The model used applies only to dimensionless ratio data such as odds ratios, relative risks, hazard ratios and meta-analyses involving aggregation of several sources of such data. With such dimensionless ratio data the expected value of the effect size is not affected by units of measurement or the size of the clinical trial. Rather than use the rather awkward term “dimensionless ratio data”, in the remainder of the paper the term “relative risk” will often be used for such data in general.

The distribution of effectiveness obtained from the Cochrane sample of relative risks together with their standard deviations is used in Bayes’ theorem to derive objective posterior probabilities. The end result is a contour graph with one axis being the relative risk and the other axis indicating the width of its confidence interval. Given particular values for these quantities from a Cochrane study, the nearest contour gives the approximate probability that the treatment studied is effective.

## Methods and Results

### Collecting the data

It turned out to be possible to download the titles of all the studies in the Cochrane Collaboration [15]. Computer programs were written to exclude all duplications and then randomly shuffle the order of the titles and to preserve this randomly ordered sequence of 8710 titles in a file. Each title in order was then input into the Cochrane search facility and in each case the first relevant statistic in the Cochrane abstract or (if the abstract gave no relevant statistics) in the body of the Cochrane review was recorded, if appropriate. The process was continued until 101 appropriate data points were obtained, each giving a relative risk together with a confidence interval. The following criteria were used to determine the appropriateness of the data:

- A data point was appropriate only if it was a dimensionless ratio point estimate such as a relative risk, together with its confidence interval.
- The log of the point estimate had to be, to within rounding error, in the middle of the log confidence interval so that the assumption that the estimate comes from a normal distribution is not contradicted. (This assumption is not true for some measures occasionally used in meta-analyses.)
- Reviews concerning new drugs or new medical devices were excluded. This criterion avoids publication biases that may be inflated by commercial pressures and, as desired, tends to over-represent Cochrane reviews of established treatments where the p-value criteria may be unnecessarily conservative [8].
- Relative risks were excluded if they referred to side effects, not the intended effect of a treatment.
- There had to be a clear implication from commonsense or basic medical knowledge that the treatment is being tried because all researchers agree about the expected direction of benefit if any (this agreement will not exclude the possibility of a counterproductive outcome). This is required because the method expects relative risks to be inverted where necessary so that an effect in the direction of anticipated benefit is given a number greater than 1.00. This inversion cannot be done objectively when there is disagreement about the direction of anticipated benefit. The treatment trials that satisfy this requirement, will often be of the form standard treatment plus new treatment versus standard treatment alone, and will often exclude trials comparing mutually exclusive treatments.

**Fig 1:**
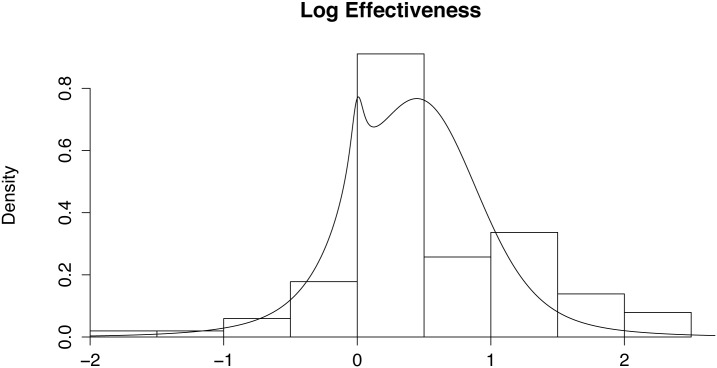
Distribution of the logarithm of the relative risks. The superimposed curve shows the fit of the model described below. Note that the model is fitted not only according to the position of the values as depicted by the histogram, but also by the accuracy with each point is measured so the visual match may not give a good impression of the fit.

A total of 321 Cochrane reviews were searched. Of these 73 were plans for a review with no actual review (“protocols”) and a further 8 were withdrawn reviews. Of the remaining 240 reviews, 29 were eliminated because they referred to new drugs or new medical devices. Of the remaining 211 reviews, 35 contained no data as a systematic review of the literature had found no randomised trials of reasonable standard. Of the remaining 176 reviews, 47 were eliminated because they contained dimensional data only. Of the remaining 129 reviews, there were 27 where it seemed using commonsense, sometimes supplemented by general medical knowledge and information in the background to the review, that there would not have been general agreement about the anticipated direction of any effect. This left 102 reviews, but there was one review which had a clear arithmetic error with the point estimate outside the range of the confidence interval and it was not possible to resolve this error using publications readily available. The final tally then was 101 usable data values, a number that seemed likely to be reasonably adequate for the purposes here in the light of previous work [8]. (The list of the 321 titles considered together with the data used from each study or else reasons for non-inclusion, is available as a supplementary document file—S1 File) A histogram of the results in terms of the logarithm of the absolute size of the relative risks, is shown in Fig 1. This figure also shows the fit of the model described below.

Each relative risk can also be classified not just by its point estimate of size, but also by the accuracy with which it is measured thereby giving the strength of evidence for an effect. The relative risks are then classified as significantly counterproductive, non-significantly negative, exactly zero effect, non-significantly positive and significantly positive. Significance here being defined by *p* < 0.05. The number of relative risks in each category were 2, 11, 2, 32 and 54 respectively.

The distribution of the standard deviations is also of interest. The data suggests that the distribution of the standard deviations is exponential. The single sample Komogorov-Smirnov (KS) test for the assumption the standard deviations come from an exponential distribution with parameter 2.935, gives a p-value of 0.132. The two sample KS test for a difference between the distribution of the standard deviations of the 54 positive “statistically significant” data points compared to the remainder gives a p-value = 0.353).

### The Standard Model

We want to estimate the intrinsic effectiveness *x* of any treatment, given a clinical trial or meta-analysis with outcome *u* subject to some uncertainties given by the standard deviation of the study *s*. To commence we assume that log relative risks chosen at random from the Cochrane Collaboration, across a disparate range of medical conditions and treatments, will have a distribution that reflects the following ideas. It is assumed that each treatment has a probability *p* of being entirely ineffective and that there is probability (1 − *p*) that the treatment has some effect. It may be anticipated that where there is an effect it will most often be positive, but occasionally, treatments that have an effect will, surprisingly and disappointingly, have a negative effect, that is, they will be counterproductive. If the treatment is effective, it is assumed that the size of this effect is given by a normal distribution with parameters *μ* and $\hat{\sigma }$. We use $\phi (x,\mu ,{\hat{\sigma }}^{2})$ to denote the density function of this normal random variable at value *x*. The distribution of intrinsic effectiveness *X* is then given by

$$\begin{array}{c} X∼p\times \delta \left(x\right)+\left(1-p\right)\times \phi \left(x,\mu ,{\hat{\sigma }}^{2}\right) \end{array}$$ (1)

where we use *p* times the Dirac *δ* function to indicate that the probability density function contains a spike of probability mass *p*, at precisely zero effectiveness.

We also assume that the variability about the true value of *x* due to the measurement error that is likely to occur in any clinical trial of finite size, is given by *Y* ∼ *N*(0, *s* ^2^) where *s* is the standard deviation. It is assumed that *s* is known accurately. We also assume that *Y* is independent of *X*. This assumption is consistent with the result of the two sample KS test given at the end of the previous section. The joint density function of *X* and *Y* is then

$$\begin{array}{c} P\left(x,y|s,\mu ,\hat{\sigma },p\right)=(p\times \delta \left(x\right)+\left(1-p\right)\times \phi \left(x,\mu ,{\hat{\sigma }}^{2}\right))\times \phi \left(y,0,s^{2}\right) \end{array}$$ (2)

Now replace the variable *Y* with the variable *U* = *Y* + *X*, where *U* is the random variable describing measured effectiveness of a treatment in a clinical study rather than the intrinsic effectiveness of a treatment, *X*. The joint probability density function of *X* and *U* is then

$$\begin{array}{ccc} P(x,u|s,\mu ,\hat{\sigma },p) & = & (p\times \delta \left(x\right)+\left(1-p\right)\times \phi \left(x,\mu ,{\hat{\sigma }}^{2}\right))\times \phi \left(u-x,0,s^{2}\right)\\ & = & p\phi \left(u-x,0,s^{2}\right)\times \delta \left(x\right)+\left(1-p\right)\times \phi \left(x,\mu ,{\hat{\sigma }}^{2}\right)\times \phi \left(u-x,0,s^{2}\right) \end{array}$$

Let *ψ* stand for the model parameters, so $\psi =\{\mu ,\hat{\sigma },p\}$ The model is then given by the density function

$$\begin{array}{c} P\left(x,u|s,\psi \right)=p\phi \left(u,0,s^{2}\right)\times \delta \left(x\right)+\left(1-p\right)\times \phi \left(x,\mu ,{\hat{\sigma }}^{2}\right)\times \phi \left(u,x,s^{2}\right) \end{array}$$ (3)

Integrating Eq (3) over all *x* then gives

$$\begin{array}{c} P\left(u|s,\psi \right)=p\phi \left(u,0,s^{2}\right)+\left(1-p\right)\phi \left(u,\mu ,{\hat{\sigma }}^{2}+s^{2}\right) \end{array}$$ (4)

The outcome of the *i* ^*th*^ clinical trial is a realisation of a random variable *U*_*i*_ with a probability density given by Eq (4) with its standard deviation *s*_*i*_ replacing *s*. The distribution of the set {*u*_*i*_} is the mixture distribution

$$\begin{array}{c} \frac{1}{101}\sum_{i=1}^{101}(p\phi \left(u_{i},0,s_{i}^{2}\right)+\left(1-p\right)\phi \left(u_{i},\mu ,{\hat{\sigma }}^{2}+s_{i}^{2}\right)) \end{array}$$ (5)

and an estimate of the parameters $\mu ,\hat{\sigma }$ and *p* can be found by maximizing the likelihood of $\prod_{i=1}^{101}(p\phi \left(u_{i},0,s_{i}^{2}\right)+\left(1-p\right)\phi \left(u_{i},\mu ,{\hat{\sigma }}^{2}+s_{i}^{2}\right))$ or equivalently by maximising $\sum_{i=1}^{101}\log(p\phi \left(u_{i},0,s_{i}^{2}\right)+\left(1-p\right)\phi \left(u_{i},\mu ,{\hat{\sigma }}^{2}+s_{i}^{2}\right))$

The Nelder and Meade simplex algorithm [16] was used to find the optimal values of the parameters $\mu ,\hat{\sigma }$ and *p*.

Non parametric confidence intervals were obtained by selecting with replacement a set of 101 data points from the original set of 101 data points and recalculating $\mu ,\hat{\sigma }$ and *p*. This process was repeated 1000 times. The results are given in Table 1 below.

**Table 1:** Parameter estimates for the standard model.

parameter	point estimate	median	95% confidence interval
*μ*	0.4775	0.4734	(0.3499, 0.6242)
$\hat{\sigma }$	0.3642	0.3593	(0.2334, 0.4657)
*p*	0.1256	0.1191	(0.000009, 0.2776)

### Main outcome

To calculate the probability (density) of an intrinsic effectiveness of *x* given a value *u* for the measured effectiveness, we use Bayes’ theorem. We require a version of Bayes’ theorem with further conditioning throughout on *s* and the parameters *ψ*. Using Eqs (3) and (4) then gives:

$$\begin{array}{ccc} P(x|u,s,\psi ) & = & \frac{P(x,u|s,\psi )}{P(u|s,\psi )}\\ & = & \frac{p\phi \left(u,0,s^{2}\right)\times \delta \left(x\right)+\left(1-p\right)\times \phi \left(x,\mu ,{\hat{\sigma }}^{2}\right)\times \phi \left(u,x,s^{2}\right)}{p\phi \left(u,0,s^{2}\right)+\left(1-p\right)\phi \left(u,\mu ,{\hat{\sigma }}^{2}+s^{2}\right)} \end{array}$$ (6)

To find the probability that the treatment is effective (that is *x* > 0) we integrate Eq (6) over all values *x* > 0 to give

$$\begin{array}{ccc} P(x>0|u,s,\psi ) & = & \frac{\left(1-p\right)\phi \left(u,\mu ,{\hat{\sigma }}^{2}+s^{2}\right)\left(1-\Phi \left(-\alpha \right)\right)}{p\phi \left(u,0,s^{2}\right)+\left(1-p\right)\phi \left(u,\mu ,{\hat{\sigma }}^{2}+s^{2}\right)}\\ & = & \frac{\left(1-p\right)\phi \left(u,\mu ,{\hat{\sigma }}^{2}+s^{2}\right)\Phi \left(\alpha \right)}{p\phi \left(u,0,s^{2}\right)+\left(1-p\right)\phi \left(u,\mu ,{\hat{\sigma }}^{2}+s^{2}\right)} \end{array}$$ (7)

where *Φ* is the cumulative distribution function of the standard normal variable and

$$\alpha =\frac{u{\hat{\sigma }}^{2}+\mu s^{2}}{\hat{\sigma }s\sqrt{{\hat{\sigma }}^{2}+s^{2}}}$$ (8)

The final value for the probability that the treatment is effective is then

$$\begin{array}{c} p_{e}=p_{\text{effective}}=P\left(x>0|u,s\right)=\int P\left(x>0|u,s,\psi \right)P\left(\psi \right)d\psi \end{array}$$

This is then approximated using the set of bootstrap estimates {*ψ*_*j*_} for the distribution of *ψ* so $p_{e}\approx \frac{1}{1000}\sum_{j=1}^{1000}P\left(x>0|u,s,\psi_{j}\right)\psi_{j}$

#### Asymptotic Behaviour

It is of interest to explore the asymptotic behaviour of Eq (7). As expected, it can be seen from Eqs (7) and (8) that if *s* → 0, *p*_*e*_ → 1 if *u* > 0 otherwise *p*_*e*_ → 0, so if effectiveness could be measured with perfect accuracy, then any value for the log of the relative risk greater than 0 would indicate certainty about effectiveness. Similarly if *s* → ∞ then whatever inaccurate point estimate of effectiveness we may also have, we only have a vanishingly small amount of information from the data about *u*, and the probability that the treatment is effective approaches the prior probability. That is it approaches the proportion of treatments with *x* > 0 as determined by fitting the model to the random sample from the Cochrane Collaboration. The model’s estimate of the proportion effective is $\left(1-p\right)\Phi (\frac{\mu }{\sigma })$ which after the bootstrap step gives 79.9%. This prior estimate of the proportion of effective treatments reviewed in the Cochrane Collaboration, is compatible with the impression obtained by noting that positive treatments outweigh negative by about 6.2 to 1. A crude calculation from this latter figure would suggest that $\frac{7.2-2}{7.2}$ or about 72% of treatments are effective.

#### Contour plots of the outcomes

It is convenient to produce a contour plot to enable at least an approximate estimation of the probability of effectiveness from a relative risk and its confidence interval. The contours correspond to a given probability of effectiveness and the axes give the relative risk and the confidence interval width. However, since a preliminary log transformation is involved in the model (and in calculating the confidence interval initially), rather than using the width of the confidence interval, it is convenient to use the width of the log confidence interval, or equivalently, the log of the ratio of the upper and lower confidence interval bounds. The log transformation also makes it convenient to use axes with relative risk and confidence bound ratio both labelled on a log scale. To produce the contour plots, the calculation of probability of effectiveness is undertaken for each point of a 500 × 500 grid of a range of values for the relative risk and the ratio of the bounds of the 95% confidence interval. The contours were then obtained using the R contour function [17].

**Fig 2:**
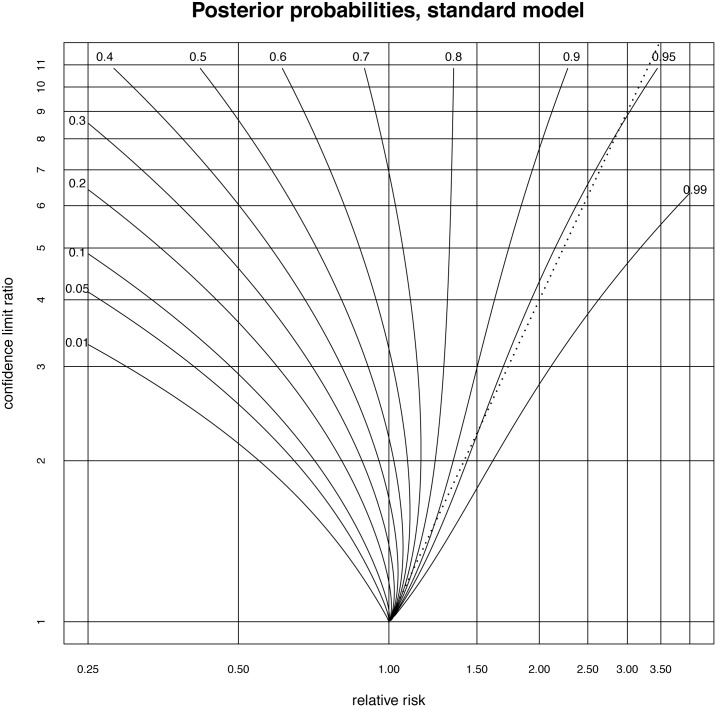
Contour plot allowing posterior probabilities of effectiveness to be estimated from the relative risk and the ratio of the bounds of the confidence interval. The dotted line close to the 0.95 posterior probability contour represents the p-value = 0.05 criterion. Note that if there is a probability of *x*% that a treatment has an effectiveness >0, then in this model the remaining (100 − *x*)% probability is not that the treatment is simply ineffective. Rather this (100 − *x*)% probability is shared out between being ineffective and being counterproductive. However, for reasons of space we omit separate contour plots for the probability of being ineffective and for being counterproductive.

The result is the plot illustrated in Fig 2. The x-axis of this plot is the relative risk, the y-axis gives the precision with which this measure is assessed in terms of the ratio of the upper to lower 95% confidence interval bounds. The contours give the probability that the treatment is effective. This plot assumes that positive results are represented by numbers larger than 1.00 and a preliminary inversion may be necessary. For example, if the relative risk of dying with the new treatment is 0.25 times that with the standard treatment, this has to be reformulated as a relative risk of 4.0 of dying by being in the control group.

The log relative risks are assumed to have a normal distribution so a relative risk of 1.5 with a confidence interval ratio of 3.0, implies log(1.5) is in the middle of the confidence interval of length log(3), so the confidence interval bounds are $\log\left(1.5\right)-\frac{1}{2}\log\left(3\right)$ and $\log\left(1.5\right)+\frac{1}{2}\log\left(3\right)$. The confidence interval ratio of 3.0 around the relative risk of 1.5, then represents a confidence interval of $\left(1.5/\sqrt{3},1.5\sqrt{3}\right)$ or (0.866, 2.598). The probability of a treatment with this relative risk and confidence interval, being effective can then be determined from the label of the contour passing through that point, so we see that the posterior probability that this treatment is effective is about 0.9.

It is of interest to compare the assessments of treatments which can be made from this diagram with assessments using p-values. We continue the example of a relative risk of 1.5 and a confidence interval bound ratio of 3.0 and therefore lying almost on the probability = 0.9 contour line. Since the 95% confidence interval represents 3.92 standard deviations, one standard deviation is $\frac{\log3}{3.92}$. Therefore the distance of the point log1.5 in standard deviations from a log relative risk of zero, is $\frac{3.92\log1.5}{\log3}$ giving the p-value to be 0.0740, so it would be conventional to not reject the null hypothesis that the experimental treatment has no effect, despite our posterior probability showing that there is a 90% chance that it is effective. More generally, consider the set of all treatments which have a p-value of 0.05. For these, the lower bound of the 95% confidence interval is 1.00 and since the geometric mean of the upper and lower bound is the relative risk, we have the upper bound being the relative risk squared. The ratio of the confidence interval bounds is then the square of the relative risk. The parabola given by the ratio of bounds of the confidence interval = the square of the relative risk is then the line that defines the p-value = 0.05 criterion. Since the contour plot is drawn using log-log axes, if we draw this line on the contour plot, it will appear as a straight line starting at the point (1.0, 1.0) and going through the point (2.0, 4.0). The p-value = 0.05 criterion is then drawn as a dotted straight line in Fig 2. It can be seen that over most of the values in the plot, the p-value criterion is very conservative compared to the probability of effectiveness, with the p-value = 0.05 criterion being close to the posterior probability 0.95 for much of the plot.

**Fig 3:**
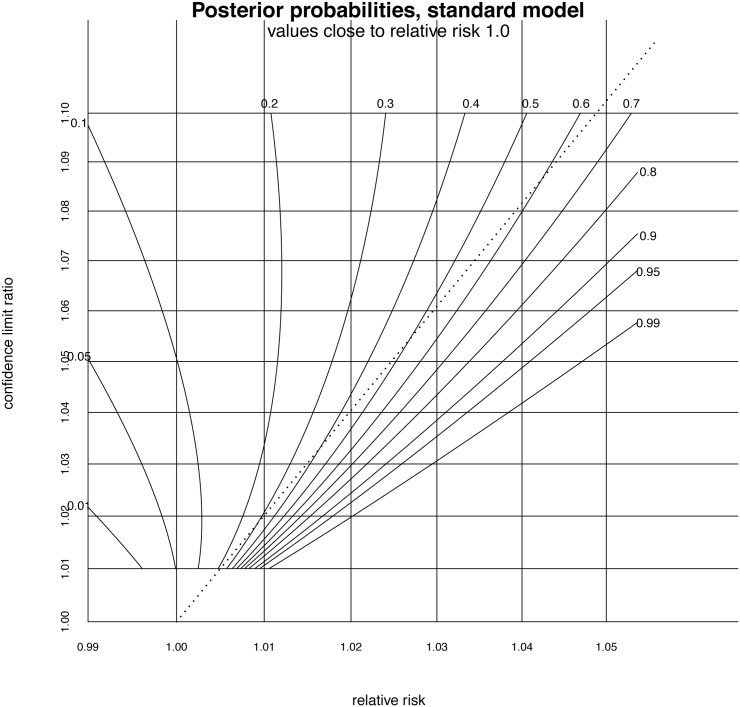
Contour plot showing posterior probabilities of effectiveness for values of relative risk close to 1.00. The p-value = 0.05 criterion displays as a dotted line on this graph. Note how this crosses the balance of probabilities contour (the posterior probability labelled 0.5), at a relative risk of 1.027.

However, for values very close to a relative risk of 1.00 the p-value criterion may be less conservative than a decision made on the balance of probabilities, that is a probability 0.5. Accordingly, assessment based on p-values is not always more conservative than assessment based on this contour plot. A magnified version of the plot near (1.0, 1.0) is given in Fig 3. It shows the line representing the p = 0.05 criterion crossing the posterior probability = 0.5 contour line at a relative risk of 1.027 with confidence interval (1.00, 1.055). Studies which yield relative risks closer to 1.00 which just pass the p = 0.05 criterion, will then be assessed as indicating effectiveness too easily compared to an assessment on the balance of probabilities.

### Model incorporating Publication Bias

Publication bias has so far not been taken into account. The extent of this problem can sometimes be quantitatively assessed by funnel plots and related techniques [18] and it can also be assessed by review of the grey literature and in the long run by medical reversals [19]. An exact measure of the extent of publication bias is not possible, but to extend this work to allow for publication bias we use an estimate for the odds ratio of publication bias of 2.78 [20]. It should be noted that this value of 2.78 is more likely to be an overestimate than an underestimate of the extent of publication bias relevant to the work here. The 2.78 estimate applies to all trials, whereas the work here deliberately excludes clinical trials involving drugs under patent where commercial pressures may add further incentive to withhold publication of results that are not statistically significant [4]. Furthermore the data here comes from the Cochrane Collaboration where particular care is taken not to overlook non-significant trials. Nevertheless, it may be reasonable to allow for the effects of publication bias and to assess its effects by using the estimate of 2.78. To account for this level of publication bias, non-parametric bootstrapping is used. In particular, random numbers are used to select 101 data points with repetition allowed from the original set of 101. However, the non-significant studies are weighted so that each is 2.78 times more likely to be selected compared to the significant data points. The parameters *μ* and $\hat{\sigma }$ and *p* are calculated and the whole process is repeated to obtain 1000 sets of values for the parameters. The results are given in Table 2 below:

**Table 2:** Parameter estimates for the model with publication bias.

parameter	mean	median	95% confidence interval
*μ*	0.4108	0.4129	(0.1989, 0.6554)
$\hat{\sigma }$	0.2997	0.3105	(0.1081, 0.4698)
*p*	0.3413	0.3579	(0.0127, 0.6141)

**Fig 4:**
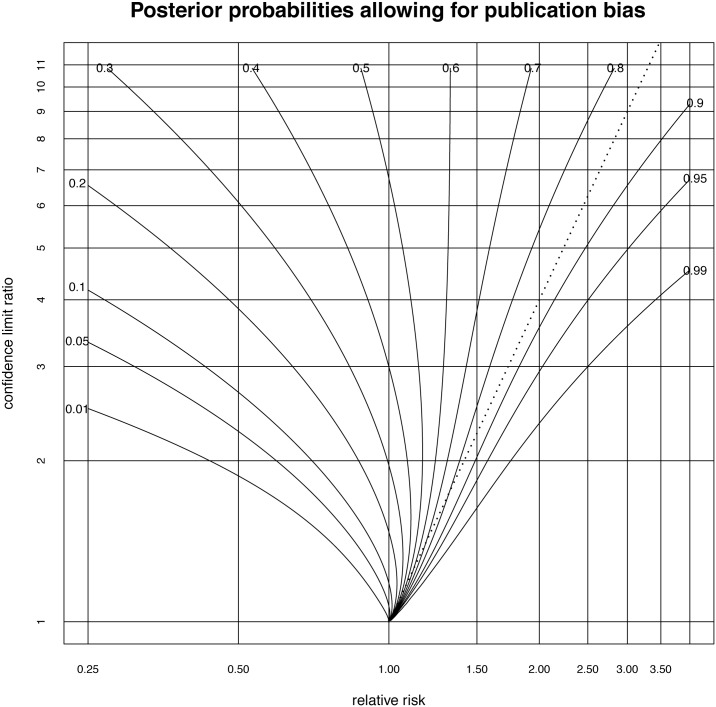
Contour plot allowing posterior probabilities of effectiveness to be estimated from the relative risk and the ratio of the bounds of the confidence interval. This plot allows for publication bias with an odds ratio of 2.78. The p-value = 0.05 criterion appears as the dotted line.

The previously described calculations to obtain a contour plot are performed with these bootstrap estimates of the parameters $\mu ,\hat{\sigma }$ and *p* and the results are displayed in Fig 4.

Again, the p-value criterion seems for the most part to be rather conservative, as the p-value = 0.05 criterion lies between the posterior probability 0.8 and 0.9 over much of the range of values shown. However, as expected, it is less conservative compared to the model without allowance for publication bias. Again for relative risks close to 1.0, the p-value = 0.05 criterion ceases to be conservative and indeed becomes too liberal compared to a decision to be made on the balance of probabilities. Here, the point at which this occurs is a relative risk of 1.081.

### An alternative simpler single peaked model

It is of interest to compare the results of the model above which makes allowance for a proportion of treatments being completely ineffective, with an alternative model. An obvious simpler model assumes that the log relative risks are normally distributed, with no spike in probability at zero effectiveness. This model has two parameters—the mean *μ* and standard deviation of intrinsic effect $\hat{\sigma }$. Following Eq (5) in the derivation of our standard model, we have the distribution of the set of log relative risks {*u*_*i*_}, being the mixture distribution

$$\begin{array}{c} \frac{1}{101}\sum_{i=1}^{101}\phi \left(u_{i},\mu ,{\hat{\sigma }}^{2}+s_{i}^{2}\right) \end{array}$$

and an estimate of the parameters is obtained maximizing the log likelihood

$$\begin{array}{c} \sum_{i=1}^{101}\log(\phi (u_{i},\mu ,{\hat{\sigma }}^{2}+s_{i}^{2})) \end{array}$$

Again, non parametric bootstrap methods are used to obtain confidence intervals and the results are given in Table 3 below.

**Table 3:** Parameter estimates for the single peaked model.

parameter	point estimate	median	95% confidence interval
*μ*	0.4167	0.4130	(0.3108, 0.5261)
$\hat{\sigma }$	0.3593	0.3554	(0.2382, 0.4599)

**Fig 5:**
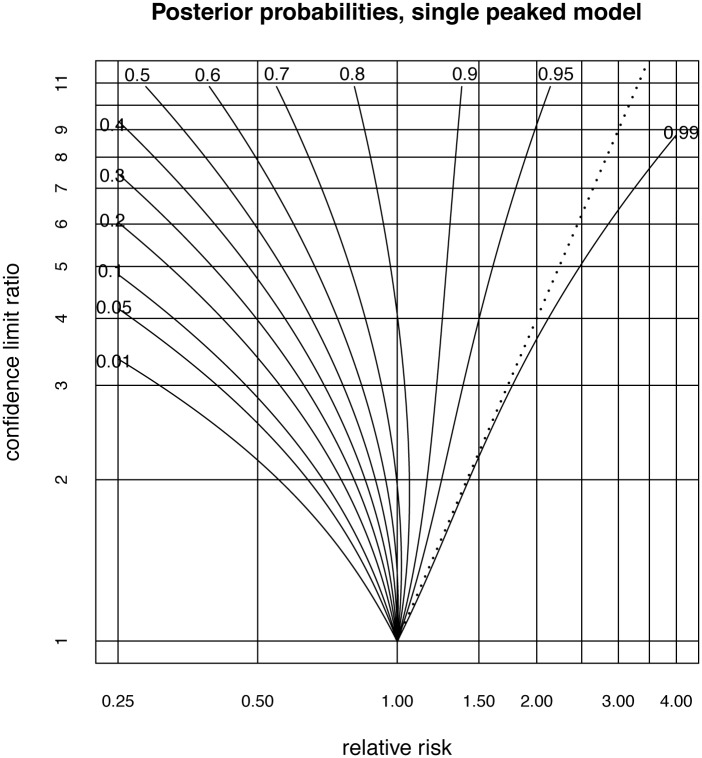
Contour plot of posterior probabilities for the single peaked model. Again the p-value = 0.05 criterion is marked by a dotted line.

The contour plot for this model is given in Fig 5. It should be noted that in this model, if a clinical trial is given a probability of *x*% of being effective, then it has a probability of (100 − *x*)% of being counterproductive, but zero chance of being simply ineffective. While there may then be an argument for giving contours in terms of net positive probability, this is not done as there will be a non-linear relationship between size of the log relative risk and the cost.

### Goodness of Fit

**Fig 6:**
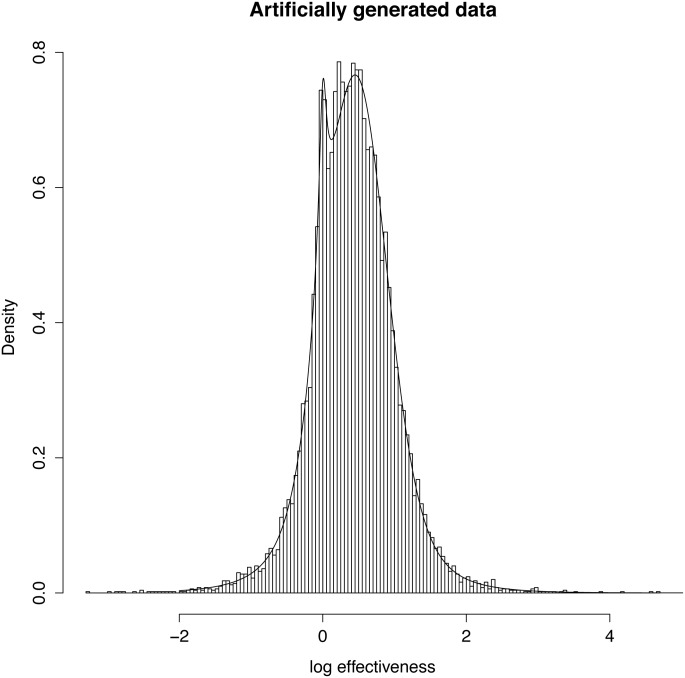
Distribution of the effectiveness of 10000 artificially generated points using the point estimates found for the standard model. The line gives the theoretical density function used to generate the data.

The method was checked by generating 10000 simulated data points using the values for the parameters $\mu ,\hat{\sigma }$ and *p* found for the standard model. The values of the parameters $\mu ,\hat{\sigma }$ and *p* were then calculated from the simulated data and the result is given in Table 4 below. A histogram of the 10000 effectiveness values is displayed in Fig 6 together with the model distribution function drawn using the parameters that generated the data.

$$\begin{array}{c} P\left(u|\left\{s_{i}\right\},\psi \right)=\frac{1}{10000}\sum_{i=1}^{10000}(p\phi \left(u,0,s_{i}^{2}\right)+\left(1-p\right)\phi \left(u,\mu ,{\hat{\sigma }}^{2}+s_{i}^{2}\right)) \end{array}$$

The calculated values of the parameters $\mu ,\hat{\sigma }$ and *p* were a close match to the values used in generating the data and the distribution function (evaluated at 8000 different values of *u*) gave an excellent match to the histogram of the generated {*u*_*i*_}

**Table 4:** Theoretical parameters used to generate a large sample and corresponding calculated parameter values from that sample.

parameter	theoretical value	model parameter estimate from analysing 10000 generated data points
*μ*	0.4775	0.4750
$\hat{\sigma }$	0.3642	0.3625
*p*	0.1256	0.1295

In effect, we have used a large parametric bootstrap sample to check the calculations of the model and have shown a good fit both in terms of parameter values and in terms of a visual fit of the model probability density to the histogram of the generated data. The fit of the model to the real data in Fig 1 is less impressive. This reflects the far smaller sample size and the fact that the model is fitting not just the size of the effectiveness but is weighting that fit by the uncertainty with which each point is measured.

To assess the goodness of fit of the real data to the model, we note that the maximum log likelihood for the standard model is -86.428 and for the alternative simpler single peaked model it is -87.977. It is noted that the standard model requires one extra parameter, but gains more than 1.5 in maximum log likelihood. The Akaike Information Criterion therefore suggests a preference for the standard model.

### Uncertainty in the data

Detailed analysis of the extent to which the posterior probability calculations could be refined by collecting more data to calibrate the model, is beyond the scope of this paper. An assessment here could require experiments with parametric bootstrap simulated data of the sort described in the previous section and experiments with non-parametric bootstrap samples as well as calibrating the model with different amounts of real data. It is noted that the upper limit to the model’s performance is obtained if the widths of the confidence intervals on the model parameters have shrunk to zero. If the model is an accurate reflection of reality, the remaining uncertainty when results from a clinical trial are assessed by the model would then be solely a reflection of the accuracy with which the relative risk is measured in the clinical trial. However, the focus of this paper was in demonstrating the method and not on the time consuming process of collecting sufficient data to obtain confidence intervals of negligible width.

Some impression of the extent to which extra data could refine the posterior probability calculation can be obtained by examining how probabilities at some selected points on the contour graph may change with various non-parametric bootstrap parameter estimates. In particular, using Fig 2, four points are chosen, two points on the posterior probability = 0.1 line at confidence interval ratios 2.0 and 5.0 and two points on the posterior probability = 0.9 line at confidence interval ratios 2.0 and 5.0. The uncertainty is estimated by 95% bootstrap confidence intervals using the 1000 non-parametric bootstrap estimates of the parameters $\mu ,\hat{\sigma }$ and *p*. Results are given in Table 5.

**Table 5:** Relation between parameter uncertainties and posterior probability calculations.

relative risk	confidence interval ratio	probability	95% conf int
0.711	2.0	0.100	(0.048, 0.217)
0.241	5.0	0.100	(0.030, 0.288)
1.345	2.0	0.900	(0.762, 0.984)
1.737	5.0	0.900	(0.795, 0.973)

The variation in the probabilities obtained by using bootstrap parameter estimates tells us that, because our information about the parameters is uncertain, we have to do the final integration step

$$\begin{array}{c} P(x>0|u,s)=\int P(x>0|u,s,\psi )P(\psi )d\psi \end{array}$$

in the model. It is also an indicator of the extent to which our estimates of posterior probability could be refined by collecting more data to calibrate the model.

In calculating the contour plot using the set of 1000 bootstrap estimations of *ψ*, we are describing a surface in three dimensions but we are using 1000 sets of three parameters to do so. It should be possible to approximate such a surface with far fewer than 3000 parameters but a good approximation with just a few parameters, has not yet been found. However, the supporting information section below, gives a link to the file of bootstrap estimations of *ψ* and some R code to enable exact calculation of posterior probability.

## Discussion

The ability to go beyond p-values and confidence intervals and obtain objective probabilities of effectiveness would seem to be an appreciable advance. However there are a number of limitations.

### Subjectivity

The aim of this work is to show the use of a Bayesian approach to clinical trial results, to enhance decision making by using non-trivial yet objective prior information. However, as discussed early in the Methods section, a small degree of subjectivity remains. There is little subjectivity in deciding whether a treatment involves a new drug and only a little more subjectivity in judging whether the researchers are agreed on the likely direction of an effect if any. Somewhat more subjectivity is needed in judging how much allowance should be made for publication bias. There is additional uncertainty about the accuracy of the model and also in any extrapolation of the use of this model to relative risks which do not (yet) appear in Cochrane, given that the model is calibrated to data currently in Cochrane.

On the other hand, much of the subjectivity discussed above may be irrelevant if the end result is invariant across a full range of uncertainty. For example, if cost considerations combined with probabilities obtained from all of the contour plots suggest that that a treatment should be implemented, then the uncertainty about which model to use and whether to allow for publication bias, is irrelevant. Of course, this can be stated with reasonable certainty only if contour plots have been prepared for all possible models that give a reasonable fit to the data and all plausible values for publication bias. However, where there is agreement using the three contour plots provided here, that a particular treatment should be regarded as worthwhile, it is reasonable to conclude that it is very likely to be worthwhile. The small amount of subjectivity that remains in using this method, will then be of little consequence.

### Relationship to p-values for relative risks near 1

All three models give a prior probability that more than half of all treatments are effective. Given the discussion in the introduction, one would then have expected the p-value = 0.05 criterion to be uniformly too conservative compared with a decision made on the balance of probabilities. However, the models which assume a spike of probability at precisely zero effectiveness show that the p-value = 0.05 criterion is not sufficiently cautious compared to a decision made on the balance of probabilities when relative risks are close to 1.00. As discussed in the section on goodness of fit, the data gives evidence in favour of the standard model of section 2.3 rather than the model of section 2.5. However there is extrapolation in use here. The data used to calibrate the model does not include relative risks just above 1.00 with very tight confidence intervals and there is certainly not enough data to conclude that the excess probability near a log relative risk of zero, is necessarily a spike at exactly zero. On the other hand, it is plausible that an appreciable proportion treatments that may be tested, do in fact target something which turns out to be entirely irrelevant to the disease process and therefore have precisely zero effectiveness, so when a treatment shows close to zero effectiveness, the balance of probabilities may well indicate that it is genuinely ineffective despite a “significant” p-value. Accordingly, more caution is needed in assessing very large trials which yield small but “significant” increases in relative risk.

The converse problem however may be more important. In most cases, the p-value = 0.05 criterion does seem unduly conservative and corresponds to objective posterior probabilities in the range 0.8 to 0.95. Such conservatism can be reasonable for new treatments with the possibility of unforeseen risks, but will often be inappropriate when low cost, established treatments are being assessed [8].

### Restricted Applicability

A major limitation of this approach is its restricted applicability. The method used here directly applies only to appropriate relative risks extracted from Cochrane as described in section 2.1 with the data being the first mentioned relative risk in a Cochrane review. However, it may be reasonable, to apply it without recalibration to any appropriate data in Cochrane, regardless of whether it was the first relative risk quoted in a review and it may also not be too unreasonable to apply it to any appropriate data likely to be included in a future Cochrane review. The method would have to be recalibrated if it were to be applied to data from other compendia of relative risk information such as a register of clinical trials of new drugs. In this case, an estimate of publication bias specific to this situation would also be required.

### Appropriate level of Effectiveness

The method described above gives only the probability that the effectiveness is any value greater than zero. Whilst, this is relevant if the cost of treatment is near zero, the probability of exceeding a particular non zero level of effectiveness, may be more relevant. For example, a more appropriate point at which to decide in favour of implementing a treatment under trial could be the amount of effectiveness at which the expected costs of Type I errors and Type II errors are equal [21].

In conventional statistical planning and analysis of clinical trials, the size of the effect that one wishes to detect is usually pre-specified, ideally with the implication that smaller effect sizes do not matter clinically (the minimal clinical important difference—MCID). In practice there is often no objective way to determine a MCID and other considerations may often determine the size of the effect to be detected. In particular, detecting say, a 1% increase in survival with a new treatment for a cancer may require many tens of thousands of subjects. Since such a large trial will seldom be feasible, a clinical trial may be designed with the power to detect only an increase in survival that is an order of magnitude greater. The specification of the effect size that the trial aims to detect, its relationship to a genuine assessment of the MCID and the appropriate statistical power of the clinical trial, is an important area in the traditional statistical approach to clinical trials that is often quite subjective. Bayesian approaches to analysing clinical trials usually are criticised by traditionalists for admitting subjectivity, so it is interesting that the methodology here could be considered to be much less subjective than the traditional approach.

Assuming an appropriate MCID has been specified, the probability that the treatment has sufficient effectiveness to warrant its implementation can then be obtained by integrating Eq (7) from the point of sufficient effectiveness onwards, rather than from zero onwards. It may also be relevant to integrate Eq (7) from −∞ to 0^−^, to take account of the possibility that a treatment could be counterproductive. More generally, a decision theory analysis would employ a cost function of effectiveness and multiply Eq (7) by this before integrating to obtain net costs.

## Practical posterior probability calculations

The files described below enable a calculation of the posterior probability from information given by a clinical trial on a relative risk and its confidence interval. The calculation uses the statistics package R [17]. It is necessary to copy and paste the 1000 sets of parameter values from S2 File (for the standard model) or S3 File (for the model allowing publication bias) into an ascii file entitled “btstrp.txt”. Then download the R function “postprob” contained in the text file S4 File and paste it into R. Then run the function postprob with the required values for *u*, the relative risk, and *cir*, the ratio of the bounds of the confidence interval.

## Supporting Information

S1 File List of Cochrane reviews selected from a random list and examined for data together with the data used from each review or the reason suitable data could not be found in that review. (DOC)

S2 File List of sets of bootstrap parameters used on the assumption that there was no publication bias. (TXT)

S3 File List of sets of bootstrap parameters used on the assumption that there was publication bias as described in the section “Model incorporating Publication Bias”. (TXT)

S4 File Text file containing the R code to give the posterior probabilities. (TXT)
