# Supplementary material for: From P-Values to Objective Probabilities in Assessing Medical Treatments
Source: PLoS One. 2015 Nov 24;10(11):e0142132. doi: 10.1371/journal.pone.0142132 (PMC4658034; doi:10.1371/journal.pone.0142132)
Supplement: S1 File — (DOC) [file pone.0142132.s001.doc]

First 116 characters of title of the Cochrane Review Reasons if excluded Result if included Direction of effect (see notes below)

========================================================================================================================================================

Venous cutdown versus the Seldinger technique for placement of totally implantable venous access ports No data (protocol only)

D-Penicillamine for preventing retinopathy of prematurity in preterm infants RR 0.32 (0.03, 3.70) +ve

Chinese herbal medicine for subfertile women with polycystic ovarian syndrome OR 2.97 (1.71, 5.17) +ve

Preventive measures for leishmaniasis No data (protocol only)

Behavioural and cognitive-behavioural interventions for outwardly-directed aggressive behaviour in people with learn Dimensional data only

Corticosteroids for acute bacterial meningitis RR 0.90 (0.80,1.01) +ve

Non-steroid agents for idiopathic pulmonary fibrosis New drugs

Intermittent iron supplementation for improving nutrition and development in children under 12 years of age RR 0.51 (0.37, 0.72) +ve

Efavirenz or nevirapine in three-drug combination therapy with two nucleoside-reverse transcriptase inhibitors for i New drug

The role of concomitant extracorporeal photopheresis for the treatment of chronic graft-versus-host disease after al No data (protocol only)

Exercise therapy for patellofemoral pain syndrome Dimensional data only

Interventions for reducing the risk of mother-to-child transmission of HIV infection RR 0.54 (0.42, 0.69) +ve

Surgical versus conservative management for odontoid fractures No data

Progestational agents for treating threatened or established preterm labour RR 0.62 (0.30,1.27) +ve

High versus low thresholds for repeat administration of surfactant in intubated preterm neonates No data (protocol only)

Nebuliser systems for drug delivery in cystic fibrosis No clear preference

Weighted vaginal cones for urinary incontinence RR 0.84 (0.76, 0.94) +ve

Screening for oesophageal cancer No data

Barrier agents for adhesion prevention after gynaecological surgery New drug/proprietary device

Mycophenolate mofetil versus methotrexate for prevention of graft-versus-host disease in people receiving allogeneic New drug?

Single dose oral lornoxicam for acute postoperative pain in adults New drug?

Vitamin D with or without calcium for treating osteoporosis in postmenopausal women No data (protocol only)

Regimens of less than six months for treating tuberculosis OR 6.1 (2.19,17.01) +ve

Pygeum africanum for benign prostatic hyperplasia RR 2.1 (1.4, 3.1) +ve

Non-steroidal anti-inflammatory drugs for acute gout RR 2.75 (1.13, 6.72) +ve

Galantamine for dementia in people with Down syndrome No data

Male circumcision for prevention of homosexual acquisition of HIV in men OR 0.86 (0.70, 1.06) +ve

Tocolytics for preterm premature rupture of membranes RR 1.67 (0.85, 3.29) –ve

Topical glyceryl trinitrate for lateral elbow pain No data (protocol only)

Acupuncture and related interventions for the treatment of symptoms associated with carpal tunnel syndrome No data (protocol only)

Antithrombotic drugs for carotid artery dissection No clear prior preference

Virtual reality training for surgical trainees in laparoscopic surgery Dimensional data only

Treatment for leiomyosarcoma and leiomyoma in children with HIV infection No data

Breathing exercises for children with asthma No data (protocol only)

Spectral entropy monitoring for adults and children undergoing general anaesthesia No data (protocol only)

Strategies for managing sexual dysfunction induced by antidepressant medication RR 11.50 (3.03, 43.67) +ve

Interventions for recurrent corneal erosions OR 5.67 (1.28, 25.0) –ve

Psychological interventions for parents of children and adolescents with chronic illness Dimensional data only

Aminopyridines for symptomatic treatment in multiple sclerosis New drug

Stapled versus handsewn methods for colorectal anastomosis surgery New device and dimensional data only

Interventions for improving adherence to ocular hypotensive therapy No quantitative data

Closed tracheal suction systems versus open tracheal suction systems for mechanically ventilated adult patients RR 0.88 (0.70, 1.12) +ve

Transtheoretical model stages of change for dietary and physical exercise modification in weight loss management for Dimensional data only

Procalcitonin, C-reactive protein, and erythrocyte sedimentation rate for the diagnosis of acute pyelonephritis in c Not ratio data

Speech and language therapy for aphasia following stroke Dimensional data only

Lumbar sympathectomy versus prostanoids for critical limb ischaemia due to non-reconstructable peripheral arterial d No data (protocol only)

Acupuncture for shoulder pain Dimensional data

Suturing versus alternative closure techniques for repair of episiotomy or second degree perineal tears No data (protocol only)

Endotracheal intubation at birth for preventing morbidity and mortality in vigorous, meconium-stained infants born a RR 1.73 (0.37, 8.12) –ve

Mobile phone messaging - a telemedicine for people with diabetes mellitus No data (protocol only)

Cholecystectomy for gallbladder polyp No data

Perioperative glycaemic control for diabetic patients undergoing surgery RR 0.46 (0.18, 1.18) +ve

Patient education for adults with rheumatoid arthritis Dimensional data only

Pre-emptive correction for haemodialysis arteriovenous access stenosis No data (protocol only)

Vaccines for women to prevent neonatal tetanus RR 0.57 (0.26, 1.24) +ve

Interventions for prevention and treatment of vulvovaginal candidiasis in women with HIV infection RR 0.68 (0.47, 0.97) +ve

Palliative radiotherapy regimens for patients with thoracic symptoms from non-small cell lung cancer RR 0.96 (0.91, 1.02) +ve

Calcium channel blockers for preventing acute tubular necrosis in kidney transplant recipients RR 0.55 (0.42, 0.73) +ve

Intravenous beta2-agonists versus intravenous aminophylline for acute asthma No clear prior preference

Whole-body vibration training for patients with neurodegenerative disease Dimensional data only

Risk of fatal and nonfatal lactic acidosis with metformin use in type 2 diabetes mellitus No ratio data

Belatacept for kidney transplant recipients New drug

Traditional Chinese medicinal herbs for induction of remission in ulcerative colitis No data

Intracervical prostaglandins for induction of labour RR 0.61 (0.47,0.79) +ve

Emergency sclerotherapy versus vasoactive drugs for bleeding oesophageal varices in cirrhotic patients Dimensional data only

Doppler ultrasound for fetal assessment in high risk pregnancies No data (withdrawn)

Prophylactic versus therapeutic amnioinfusion for oligohydramnios in labour RR 1.29 (0.60, 2.74) –ve

Homocysteine-lowering interventions for preventing cardiovascular events RR 1.02 (0.95, 1.10) –ve

Thioridazine for dementia OR 4.91 (3.21, 7.50) +ve

Stem cell therapy for chronic ischaemic heart disease and congestive heart failure RR 0.28 (0.14, 0.53) +ve

Intravenous immunoglobulin for treating sepsis, severe sepsis and septic shock RR 0.81 (0.70, 0.93) +ve

Laparoscopic versus Open surgery for small bowel Crohn's disease No clear prior preference

Support surfaces for pressure ulcer prevention RR 0.40 (0.21, 0.74) +ve

Surgery for treating hip impingement (femoroacetabular impingement) No data

Hormone replacement therapy for women with type 1 diabetes mellitus No data

Scapular fixation in muscular dystrophy No data

Vitamin E supplementation for adults with diabetes mellitus No data (protocol only)

Interventions to implement prevention in primary care No data (withdrawn)

Surgical versus non-surgical interventions in patients with adolescent idiopathic scoliosis No data (protocol only)

Pleurodesis for malignant pleural effusions No data (withdrawn)

Citrate salts for preventing and treating kidney stones No data (protocol only)

Effects of interventions aimed at changing the length of primary care physicians' consultation Dimensional data only

Pre-operative biliary drainage for obstructive jaundice RR 1.12 (0.73, 1.71) –ve

Beta-blockers for unstable angina No data (protocol only)

Long-acting muscarinic antagonists (LAMA) added to inhaled corticosteroids (ICS) versus addition of long-acting beta No data (protocol only)

Monitoring of stimulated cycles in assisted reproduction (IVF and ICSI) OR 1.10 (0.79, 1.54) +ve

Androgens (dehydroepiandrosterone or testosterone) in women undergoing assisted reproduction No data (protocol only)

Acupuncture for acute management and rehabilitation of traumatic brain injury OR 3.50 (1.07, 11.48) +ve

Support during pregnancy for women at increased risk of low birthweight babies RR 0.79 (0.68, 0.92) +ve

Antiviral interventions for liver transplant patients with recurrent graft infection due to hepatitis C virus No data on primary outcomes

Oral vaccines for preventing cholera No ratio data

Acupuncture for adults with type 2 diabetes mellitus No data (protocol only)

Irrigants for non-surgical root canal treatment in mature permanent teeth No accessible adequate data (in abstract or article, references locked)

Oral antihistamine-decongestant-analgesic combinations for the common cold OR 0.27 (0.15, 0.50) +ve

Cognitive reframing for carers of people with dementia Dimensional data only

Human menopausal gonadotropin versus recombinant follicle stimulation hormone for ovarian stimulation in assisted re No data (withdrawn)

Vitamin B6 for cognition Dimensional data only

Osteotomy, compression and other modifications of surgical techniques for internal fixation of extracapsular hip fra Multiple comparisons with no clear prior preference & no data

Electromechanical and robot-assisted arm training for improving generic activities of daily living, arm function, an Dimensional data only

Touch therapies for pain relief in adults No data (out of date)

Platelet count, spleen length, and platelet count/spleen length ratio for the diagnosis of oesophageal varices in pa No data (protocol only)

Pimozide for tics in Tourette's syndrome Dimensional data only

Maintenance treatments for opiate -dependent adolescents RR 0.97 (0.78, 1.22) +ve

Prophylactic vitamin K for vitamin K deficiency bleeding in neonates RR 0.73 (0.56, 0.96) +ve

Interpersonal, cognitive analytic and other integrative therapies versus treatment as usual for depression No data (protocol only)

Lifestyle interventions for the treatment of urinary incontinence in adults No data (protocol only)

Antibody induction therapy for lung transplant recipients RR 0.99 (0.69, 1.41) +ve

Open versus laparoscopic (assisted) ileo pouch anal anastomosis for ulcerative colitis and familial adenomatous poly No ratio data

Psychological, social and welfare interventions for psychological health and well-being of torture survivors No ratio data

Antiplatelet therapy for aneurysmal subarachnoid haemorrhage RR 0.79 (0.62, 1.01) +ve

Negative pressure wound therapy for managing the open abdomen after midline laparotomy No data (protocol only)

Calcium channel blockers for acute traumatic brain injury OR 0.91 (0.70,1.16) +ve

Pharmacological treatment for Attention Deficit Hyperactivity Disorder (ADHD) in children with comorbid tic disorder No clear prior preference

Extra fluids for breastfeeding mothers for increasing milk production No data

Acupuncture for hypoxic ischemic encephalopathy in neonates No data

Chemotherapy and supportive care versus supportive care alone for advanced non-small cell lung cancer HR 0.77 (0.71,0.83) +ve

Interventions for managing absenteeism among health workers No data (withdrawn)

Deep transverse friction massage for treating lateral elbow or lateral knee tendinitis RR 3.27 (0.44,24.34) +ve

Interventions for the treatment of Morton's neuroma RR 1.79 (1.25, 2.55) +ve

Interventions to improve transition of care for adolescents from paediatric services to adult services No data (protocol only)

Interventions for preventing infectious complications in haemodialysis patients with central venous catheters RR 0.17 (0.07, 0.43) +ve

Quetiapine for schizophrenia New drug

Orthotic devices for the treatment of tennis elbow No ratio data for trials with clear prior preference

Protein containing synthetic surfactant versus animal derived surfactant extract for the prevention and treatment of New drug

Care delivery and self-management strategies for adults with epilepsy Dimensional data only

Betahistine for Ménière's disease or syndrome RR 1.17 (0.86, 1.58) +ve

Inspiratory muscle training for asthma Dimensional data only

Fingolimod for relapsing remitting multiple sclerosis New drug

Injection therapy for subacute and chronic low-back pain RR 1.13 (0.78, 1.62) +ve

Cervical assessment by ultrasound for preventing preterm delivery RR 0.59 (0.26, 1.32) +ve

Radiofrequency denervation for chronic neck pain No data (protocol only)

Single or double-level anterior interbody fusion techniques for cervical degenerative disc disease No prior preference & no data on primary outcome

Interventions for prevention of giant retinal tear in the fellow eye No data

Magnetic resonance imaging, computer tomography scan, and oesophagography for the diagnosis of oesophageal varices No data (protocol only)

Chemotherapy for newly diagnosed inoperable brain metastases from non-small cell lung cancer No data (protocol only)

Magnesium sulphate versus diazepam for eclampsia No prior preference

Self-management education for adults with epilepsy No data - withdrawn

Retinoic acid post consolidation therapy for high-risk neuroblastoma patients treated with autologous hematopoietic HR 0.87 (0.46, 1.63) +ve

Ribavirin plus interferon versus interferon for chronic hepatitis C RR 0.72 (0.68, 0.75) +ve

Yoga for asthma No data (protocol only)

Manipulative therapies for infantile colic OR 6.33 (1.54, 26.00) +ve

Occupational therapy for rheumatoid arthritis Dimensional data only

Effects of communicating DNA-based disease risk estimates on risk-reducing behaviours OR 1.35 (0.76, 2.39) +ve

Non-pharmacological interventions for cognitive impairment due to systemic cancer treatment No data (protocol only)

Acupuncture for treatment-related side effects in women with breast cancer No data (protocol only)

Subcutaneous rapid-acting insulin analogues for diabetic ketoacidosis No data (protocol only) & new drugs

Sleep positioning for children with cerebral palsy No data (protocol only)

Positive end expiratory pressure for preterm infants requiring conventional mechanical ventilation for respiratory No prior preference

Aromatherapy for pain management in labour RR 1.04 (0.48, 2.28) –ve

Corticosteroids for parasitic eosinophilic meningitis No dimensionless data

Teriflunomide for multiple sclerosis New drug

Strategies to increase participant recruitment to research studies by healthcare professionals No data (protocol only)

Neuraxial anaesthesia for lower-limb revascularization OR 0.89 (0.38,2.07) +ve

Percutaneous transluminal balloon angioplasty and stenting for carotid artery stenosis No prior preference

Dietary exclusions for established atopic eczema RR 1.51 (1.07, 2.11) +ve

Continuous passive motion following total knee arthroplasty in people with arthritis Dimensional data only

Pharmacological interventions for treating dyslipidemia in patients with HIV infection No data (protocol only)

Chlorpromazine dose for people with schizophrenia No prior preference

Phosphodiesterase inhibitors for erectile dysfunction in patients with diabetes mellitus New drug

Chlorpromazine versus placebo for schizophrenia RR 0.65 (0.47, 0.90) +ve

Intravenous immunoglobulin to prevent relapses during pregnancy and postpartum in multiple sclerosis No data (protocol only)

Antithrombin for respiratory distress syndrome in preterm infants RR 2.67 (0.72, 9.83) –ve

Knowledge translation strategies for facilitating evidence-informed public health decision making among managers No data (protocol)

Pelvic floor muscle training versus other active treatments for urinary incontinence in women No data (protocol)

Mini-Cog for the diagnosis of Alzheimer’s disease dementia and other dementias within a secondary care setting No data (protocol)

Male involvement for increasing the effectiveness of prevention of mother-to-child HIV transmission (PMTCT) program No useful data

Pharmacological interventions for the treatment of anxiety disorders in chronic obstructive pulmonary disease Dimensional data only

Conservative management for postprostatectomy urinary incontinence RR 0.85 (0.60, 1.22) +ve

Indomethacin for asymptomatic patent ductus arteriosus in preterm infants RR 0.36 (0.19, 0.68) +ve

Antibiotic treatment for Clostridium difficile-associated diarrhea in adults RR 9.00 (1.24,65.16) +ve

Polyunsaturated fatty acid supplementation for schizophrenia RR 0.73 (0.54, 1.00) +ve

Physical interventions to interrupt or reduce the spread of respiratory viruses Arithmetic error

Antiviral prophylaxis for the prevention of chronic hepatitis C virus in patients undergoing liver transplantation RR 1.31 (0.41, 4.19) –ve

Interventions for fatigue in Parkinson's disease No data (protocol)

Inhaled steroids and risk of pneumonia for chronic obstructive pulmonary disease OR 1.78 (1.50,2.12) +ve

Co-enzyme Q10 supplementation for the primary prevention of cardiovascular disease Dimensional data only

Leukotriene inhibitors for bronchiolitis in infants and young children Dimensional data only & new drug

Semen preparation techniques for intrauterine insemination No clear prior preference

Ofatumumab for rheumatoid arthritis No data (protocol) & new drug

Minimally invasive surgery versus radiotherapy/chemoradiotherapy for early-stage oropharyngeal carcinoma No data (protocol)

Olanzapine alone or in combination for acute mania Dimensional data and new drug

Protocolized versus non-protocolized weaning for reducing the duration of invasive mechanical ventilation in newborn No data (protocol)

Erythropoiesis-stimulating agents for anaemia in adults with chronic kidney disease: a network meta-analysis New drugs

Pharmacologic therapies for adults with acute lung injury and acute respiratory distress syndrome New drug

Low- versus high-dose of radioiodine for thyroid remnant ablation in differentiated thyroid carcinoma No data (protocol)

Postoperative tamoxifen for ductal carcinoma in situ HR 0.75 (0.61,0.92) +ve

Bendamustine for patients with indolent B cell lymphoid malignancies including chronic lymphocytic leukaemia New drug

Biomarkers for assessing disease activity in inflammatory bowel disease No data (protocol)

Sulpiride dose for schizophrenia No data (protocol)

Interventions in the management of serum lipids for preventing stroke recurrence OR 0.88 (0.77, 1.00) +ve

Education programmes for people with diabetic kidney disease RR 1.63 (1.01, 2.63) +ve

Somatostatin analogues for thyroid associated ophthalmopathy No data (protocol)

Creatine for Parkinson's disease Dimensional data only

Acupuncture for amblyopia in children No data (protocol)

Closed reduction methods for treating distal radial fractures in adults No prior preference

Psychological interventions for depression in heart failure No data

Psychological and pharmacological interventions for depression in patients with diabetes mellitus and depression OR 2.88 (1.58, 5.25) +ve

Surgical versus endoscopic treatment of bile duct stones No prior preference

Skin patch and vaginal ring versus combined oral contraceptives for contraception New device

Token economy for schizophrenia Dimensional data

Psychosocial interventions for women enrolled in alcohol treatment during pregnancy No data

Exercise for depression Dimensional data only

Carbonic anhydrase inhibitors for hypercapnic ventilatory failure in chronic obstructive pulmonary disease Dimensional data only

Central action beta-blockers versus placebo for neuroleptic-induced acute akathisia RR 1.04 (0.59, 1.83) –ve

Prostaglandins versus oxytocin for prelabour rupture of membranes at term Out of date

Amantadine for fatigue in multiple sclerosis No ratio data

Mechanical dilatation of the cervix at non-labour caesarean section for reducing postoperative morbidity RR 1.07 (0.52,2.21) +ve

Pharmacotherapy augmentation strategies in treatment-resistant anxiety disorders RR 3.16 (1.08, 9.23) +ve

Cranberries for preventing urinary tract infections RR 0.86 (0.71, 1.04) +ve

Hyperbaric oxygen therapy for multiple sclerosis OR 0.33 (0.09, 1.18) +ve

Perioperative dexmedetomidine for acute pain after abdominal surgery in adults Protocol and new drug

Corticosteroids as standalone or add-on treatment for sore throat RR 3.16 (1.97, 5.08) +ve

Natalizumab for relapsing remitting multiple sclerosis New drug

Tissue adhesives for traumatic lacerations in children and adults No clear prior preference

Chinese medicinal herbs for chronic hepatitis B RR 3.35 (1.49, 7.56) +ve

Angioplasty versus stenting for subclavian artery stenosis No data

Pioglitazone for type 2 diabetes mellitus New drug

Maternal oxygen administration for fetal distress RR 3.51 (1.34, 9.19) –ve

Autologous chondrocyte implantation for full thickness articular cartilage defects of the knee RR 1.27 (1.02, 1.59) +ve

Systemic corticosteroid regimens for prevention of bronchopulmonary dysplasia in preterm infants No data (protocol)

Cot-nursing versus incubator care for preterm infants RR 1.48 (1.04, 2.09) +ve

Interventions to reduce risky sexual behaviour for preventing HIV infection in workers in occupational settings RR 0.68 (0.48, 0.96) +ve

Topical glyceryl trinitrate for rotator cuff disease RR 1.91 (1.04, 3.50) +ve

Melatonin for the treatment of dementia Dimensional data only

Active placebos versus antidepressants for depression Dimensional data only

Glutamine for induction of remission in Crohn's disease No data (protocol)

Over-the-counter (OTC) medications to reduce cough as an adjunct to antibiotics for acute pneumonia in children and OR 0.36 (0.16, 0.77) +ve

Topical silver for preventing wound infection Dimensional data only

Phyllanthus species for chronic hepatitis B virus infection RR 0.95 (0.73, 1.25) +ve

Methotrexate for treating rheumatoid arthritis RR 3.03 (1.53, 5.98) +ve

Oral non-steroidal anti-inflammatory drugs versus other oral analgesic agents for acute soft tissue injury No data (protocol)

Low pressure versus standard pressure pneumoperitoneum in laparoscopic cholecystectomy RR 3.00 (0.14, 65.90) +ve

Psychological interventions for antisocial personality disorder OR 8.56 (1.33, 54.95) +ve

Techniques for preparation prior to embryo transfer No prior preference

Roselle for hypertension in adults No data

Effects of preventive oral supplementation with iron or iron with folic acid for women following childbirth No data (protocol)

Multi-professional simulation-based team training in obstetric emergencies for improving patient outcomes No data (protocol)

Capsule endoscopy for the diagnosis of oesophageal varices in people with chronic liver disease or portal vein No ratio data

Interventions for preventing recurrent urinary tract infection during pregnancy RR 0.89 (0.31, 2.53) +ve

Oestrogens for treatment or prevention of pelvic organ prolapse in postmenopausal women OR 0.50 (0.31,0.81) +ve

Health, not weight loss, focused programmes versus conventional weight loss programmes for cardiovascular risk No data (protocol)

Combination formoterol and budesonide as maintenance and reliever therapy versus current best practice New device

Surgical interventions for pharyngeal pouch No data

Valproate for schizophrenia RR 0.85 (0.69, 1.04) +ve

Fixed-dose combination therapy for the prevention of cardiovascular disease RR 1.26 (0.67, 2.38) –ve

Image guided surgery for the resection of brain tumours RR 0.13 (0.02, 0.96) +ve

Prolonged thromboprophylaxis with Low Molecular Weight heparin for abdominal or pelvic surgery RR 0.41 (0.26, 0.63) +ve

Shouldice technique versus other open techniques for inguinal hernia repair No clear prior preference

Thrombophilia testing for prevention of recurrent venous thromboembolism No data

Interventions for fetal immobilisation during fetal surgery and invasive procedures No data (protocol)

Milrinone for the treatment of cardiac dysfunction in neonates No data (protocol) and new drug

Laparoscopic versus open transhiatal oesophagectomy for oesophageal cancer No data (protocol)

Perioperative enhanced recovery programmes for gynaecological cancer patients No data

Needle aspiration versus incision and drainage for the treatment of peritonsillar abscess No data (protocol)

Gloves, extra gloves or special types of gloves for preventing percutaneous exposure injuries in healthcare RR 0.29 (0.23, 0.37) +ve

Drug treatment for facioscapulohumeral muscular dystrophy Dimensional data only

Complementary and miscellaneous interventions for nocturnal enuresis in children RR 0.67 (0.48, 0.94) +ve

Tissue adhesives for closure of surgical incisions No clear prior preference

Telephone communication of HIV testing results for improving knowledge of HIV infection status No clear ratio data

Peri-implantation glucocorticoid administration for assisted reproductive technology cycles OR 1.21 (0.67, 2.19) +ve

Excitatory amino acid antagonists for acute stroke New drug

Leukotriene receptor antagonists for non-cystic fibrosis bronchiectasis New drug

Atomoxetine for schizophrenia New drug and protocol

Corticosteroids as adjunctive therapy in the treatment of influenza No data (protocol)

Selenium for alleviating the side effects of chemotherapy, radiotherapy and surgery in cancer patients No ratio data

Abdominal lift for laparoscopic cholecystectomy RR 1.00 ((0.17, 5.77) –/+

Probiotic fermented milk or isolated probiotic bacteria for primary prevention of cardiovascular disease in adults No data (protocol)

Sphincterotomy for biliary sphincter of Oddi dysfunction OR 6.52 (1.38,30.71) +ve

Colloids versus crystalloids for fluid resuscitation in critically ill patients RR 1.01 (0.93, 1.10) –ve

Email for the coordination of healthcare appointments and attendance reminders No data

Urinary alkalinisation for acute chlorophenoxy herbicide poisoning No data

Thalidomide and thalidomide analogues for maintenance of remission in Crohn's disease No data

Antibiotics for community-acquired lower respiratory tract infections secondary to Mycoplasma pneumoniae in children No ratio data

Antibiotics and antiseptics for venous leg ulcers Multiple comparisons with no prior preference

Wound drainage following groin dissection for malignant disease in adults No data

Pet allergen control measures for allergic asthma in children and adults OR 0.15 (0.03, 0.92) +ve

Group-based parent-training programmes for improving emotional and behavioural adjustment in children from birth Dimensional data only

Monoaminergic agonists for acute traumatic brain injury No data

Percutaneous ethanol injection or percutaneous acetic acid injection for early hepatocellular carcinoma No clear prior preference

Emergency ultrasound-based algorithms for diagnosing blunt abdominal trauma RR 1.00 (0.50, 2.00) –/+

Dressings for superficial and partial thickness burns No clear prior preference amongst multiple comparisons

Manual ventilation devices for neonatal resuscitation No data (protocol)

Pressure modification for improving usage of continuous positive airway pressure machines in adults with obstructive Dimensional data only

Tolerability of selective cyclooxygenase 2 inhibitors used for the treatment of rheumatological manifestations of New drug

Opioid agonist treatment for pharmaceutical opioid dependent people No data (protocol)

Drugs versus placebo for dysthymia RR 0.68 (0.59, 0.78) +ve

Rituximab for thyroid-associated ophthalmopathy New drug and no data

Interventions to prevent misconduct and promote integrity in research and publication No data (protocol)

Tamoxifen for relapse of ovarian cancer No data

Ambulatory versus conventional methods for monitoring blood pressure during pregnancy No data

Optimal duration of exclusive breastfeeding No clear prior preference

Surgical interventions for the early management of Bell's palsy No clear prior preference

Blood pressure lowering efficacy of alpha blockers for primary hypertension No ratio data

Interventions for improving communication with children and adolescents about a family member's cancer No ratio data

Follow-up protocols for women with cervical cancer after primary treatment No data

Food fortification with calcium and vitamin D: impact on health outcomes No data (protocol)

Steroid hormones for contraception in men No prior preferences amongst multiple comparisons

Interventions for treating phosphorus burns No data

Behavioural and cognitive behavioural therapy for obsessive compulsive disorder in children and adolescents RR 0.14 (0.05, 0.38) +ve

Discharge planning from hospital to home RR 0.82 (0.73, 0.92) +ve

Hepatitis B immune globulin for preventing hepatitis B recurrence after liver transplantation No data (protocol)

Short acting insulin analogues versus regular human insulin in patients with diabetes mellitus New drug and dimensional data

Simple tests to screen for diabetic peripheral neuropathy No data (protocol)

Intravenous fluids for abdominal aortic surgery No clear prior preference and multiple comparisons

Strategies for the withdrawal of nasal continuous positive airway pressure (NCPAP) in preterm infants No clear prior preference

Exercise for improving balance in older people Dimensional data only

Psychological interventions for women with metastatic breast cancer OR 1.46 (1.07, 1.99) +ve

Polyunsaturated fatty acid supplementation in infancy for the prevention of allergy and food hypersensitivity No data (protocol)

Physical training for McArdle disease No data

Lactulose versus Polyethylene Glycol for Chronic Constipation No clear prior preference

Antibiotics for community-acquired pneumonia in adult outpatients No prior preference

Adrenaline for prevention of morbidity and mortality in preterm infants with cardiovascular compromise No data

Non-pharmacological management of infant and young child procedural pain Dimensional data only

Deep versus shallow suction of endotracheal tubes in ventilated neonates and young infants No prior preference

Activity restriction for short-term and medium-term outcomes following mild traumatic brain injury No data (protocol)

Interventions for the reduction of shoulder pain following gynaecological laparoscopic procedures No data (protocol)

Probiotics for preventing urinary tract infections in adults and children No data (protocol)

Pharmacological interventions for hypertensive emergencies RR 0.72 (0.31, 1.72) +ve

Duloxetine versus other anti-depressive agents for depression New drug

Corticosteroids for the treatment of Kawasaki disease in children No data (protocol)

Duration of antibacterial treatment for uncomplicated urinary tract infection in women RR 1.06 (0.88, 1.28) +ve

Note 1: Except for the 2 cases where the RR is exactly 1.00, the entry under the "Direction of Effect" heading is used to determine if the value is to be inverted. Values are listed positive (+ve) or negative (–ve) according to whether the effect is in the direction anticipated. Positive effects less than 1.00 and negative effects greater than 1.00 are inverted before presentation in Figure 1 and subsequent processing. All the data used in the paper then is >1 if the effect is in the direction that would be anticipated assuming there was a non-zero effect and is <1 if it is in the opposite direction to that which would be anticipated if there were to be any effect.

Note 2: Care is sometimes required with double negative situations. For example, a trial with reasonable power, may examine whether a treatment maybe redundant. A small and non-significant increase in risk may then be interpreted as positive for the hypothesis that the treatment is redundant, but it is also positive in that it adds weak evidence that any change in risk is an increase in the direction that would be anticipated, assuming there were to be any effect.
